# COVID-19: a National Survey on perceived level of knowledge, attitude and practice among frontline healthcare Workers in Nepal

**DOI:** 10.1186/s12889-020-10025-8

**Published:** 2020-12-14

**Authors:** Nira Tamang, Punam Rai, Siddhartha Dhungana, Binod Sherchan, Bikash Shah, Prajjwal Pyakurel, Saroj Rai

**Affiliations:** 1 Department of Nursing, Norvic International Hospital, Kathmandu, Nepal; 2 Kopila Dental Care, Kathmandu, Nepal; 3 Department of Orthopedics and Trauma Surgery, National Trauma Center, National Academy of Medical Sciences, Mahankal, Kathmandu, 44600 Nepal; 4 B.P. Koirala Institute of Health Sciences, Dharan, Nepal

**Keywords:** Frontline healthcare workers, COVID-19, Coronavirus disease, Knowledge, Attitude, Practice

## Abstract

**Background:**

The aim of this study was to determine the knowledge, attitude and practice (KAP) regarding the COVID-19 among frontline healthcare workers (F-HCWs) working at different hospitals in Nepal and to identify the factors significantly associated with KAP.

**Methods:**

We used a web-based survey, and a convenience sampling method was adopted to collect data from 603 F-HCWs working at different hospitals in Nepal during the first week of June 2020. A self-administered questionnaire was utilized to assess the KAP perceived by the F-HCWs. It was divided into 4-parts consisting of 30-items, demographic characteristics (10-items), knowledge (10-items), attitude (5-items), and practice (5-items). It consisted of both multiple-choice questions and Likert scale items questionnaire.

**Results:**

Among the participants, 76% reported adequate knowledge, 54.7% reported positive attitude, and 78.9% reported appropriate practice. Statistically significant differences regarding the perceived level of knowledge among F-HCWs were observed among independent variables, including age, gender, level of education, marital status, profession, work experience, source of information, infection prevention and control (IPC) training, and online course(*p* < 0.05). Similarly, statistically significant differences regarding the attitude among F-HCWs were observed among independent variables, including age, gender, level of education, profession, and online course(*p* < 0.05). Moreover, only 2-independent variables, including the profession and online course, showed statistically significant differences with practice(*p* < 0.05). Pearson correlation analysis showed a significant association between knowledge, attitude and practice at the level of *p* = 0.01. The factors significantly associated with adequate knowledge were male gender, nurse and doctor, websites and IPC training. Similarly, factors significantly associated with positive attitude were online course related to COVID-19 only. Moreover, factors significantly associated with appropriate practice were master’s degree or above and online course related to COVID-19.

**Conclusions:**

F-HCWs reported adequate overall knowledge with a positive attitude and adopted the appropriate practice. The experienced F-HCWs with higher education and who received IPC training and online course regarding COVID-19 had better KAP. So, the stakeholders must arrange the educational programs and training for F-HCWs for better preparedness tackling with COVID-19.

## Background

The Coronavirus Disease 2019 (COVID-19) is recently identified as a fatal respiratory problem caused by the Novel Coronavirus subtype SARS-CoV-2 (Severe Acute Respiratory Syndrome Coronavirus 2) [1]. On the last of December 2019, a cluster of pneumonia cases appeared in Wuhan, a highly populated city in Central China, where more than 11 million population reside [2]. The disease is highly contagious and characterized by fever, cough, dyspnoea, fatigue, myalgia, and anosmia [3]. On chest computed tomographic (CT) scan, bilateral lung infiltration with ground glass appearance is evident [4]. The World Health Organization (WHO) stated the Chinese outbreak of Novel coronavirus as a public health emergency on January 30 [5] and named COVID-19 on February 11, 2020 [6]. The disease rapidly spread over 114 countries and infected more than 118,000 people, including 4291 deaths, so the WHO declared COVID-19 as a global pandemic on March 11, 2020 [2]. On January 13, 2020, the first case was detected in Nepal, a 32-year-old man studying at Wuhan, who returned to Nepal for winter vacation [7]. He went to the hospital for a cough. For a positive history of travel from the COVID-19 epicenter, and he was investigated for COVID-19. A throat swab was taken and sent to Hongkong for real-time reverse transcription-polymerase chain reaction (RT-PCR) and tested positive for COVID-19 [7].

Frontline healthcare workers (F-HCWs), including doctors, nurses, and paramedics, are prone to get infected [8]. A report on the Pan American Health Organization (PAHO) declared that nearly 570,000 COVID-19 infections and at least 2500 death among F-HCWs across the region by September 2, 2020 [9]. Similarly, India reported 87,176 contaminations and 573 deaths among F-HCWs by August 29, 2020. However, there still exists a lack of official data reported by the responsible bodies [10]. That has put the F-HCWs at a higher risk of infection, further leading to increased risk to the patients. Meanwhile, Nepal also reported the 1st COVID-19 case among F-HCWs (a nurse) on May 12, 2020, which has increased to 986 F-HCWs with at least 2 deaths on October 13, 2020.

The knowledge, attitude and practice (KAP) regarding pandemic among healthcare workers have been reported differently in different studies [11–15]. COVID-19 infection among local F-HCWs was common at the initial stage of the disease outbreak in Wuhan. However, the infection rate was reduced to nil among the F-HCWs, who were deployed from different provinces to combat COVID-19—the reason they reported having infections at the initial stage was negligence and lack of knowledge [16]. Zhang et al. [13] also reported that the lack of knowledge among F-HCWs is the causative factor for disease infection & transmission. A similar study from Greece found that a high level of knowledge among healthcare workers was significantly associated with a positive attitude and practice towards preventive health measures [17].

The aim of this study was to determine the knowledge, attitude and practice regarding the COVID-19 among frontline healthcare workers working at different hospitals in Nepal and to identify the factors significantly associated with KAP.

## Methods

### Study design and setting

It is a cross-sectional web-based survey, and a convenience sampling method was adopted. Data were collected using a self-administered questionnaire from the F-HCWs working at different hospitals of all seven provinces of Nepal, including private hospitals, government hospitals, and others (semi-government or university hospitals). These hospitals are tertiary care hospitals, where COVID-19 patients are currently being treated.

### Study participants

Registered F-HCWs, including doctors, nurses, and paramedics, working at different hospitals as F-HCWs for COVID-19, were included in this survey. Paramedics, including health assistant (HA) and community medicine assistant (CMA), are trained to provide emergency medical care. Medical students and healthcare workers currently not working in clinical settings or previously participated in a similar study regarding COVID-19 were excluded.

#### Sample size calculation

The sample size was calculated from a known population with a formula,

$$ \mathrm{Sample}\ \mathrm{size}\ \left(\mathrm{n}\right)=\frac{\frac{Z^2\mathrm{xp}\ \left(1-\mathrm{p}\right)}{e^2}}{1+\left(\frac{Z^2\mathrm{xp}\ \left(1-\mathrm{p}\right)}{e^2N}\right)} $$

Where the population size (N) is 209,552, including registered doctors, nurses, and paramedics in their respective professional councils. Confidence Level is 95%, population proportion (*p*) is 0.5, margin of error (*e*) is 0.05 (5%), alpha divided by 2 (1-Confidence Level) is 0.025 and *Z*-score is 1.96. From the above formula, the minimum required sample size (n) calculated was 384.

### Data collection procedure

The nation had strict lockdown to mitigate disease transmission, so we used a web-based survey to collect data during the first week of June 2020. Potential participants were approached via telephone calls, e-mails, and social media groups, including Viber, WhatsApp, Facebook, etc. A Google Form questionnaire was sent to each of the participants via their e-mails and social media and requested them to fill up and submit. The first page of the Google Form included the consent form that explained the research project overview and participant’s confidentiality, making sure that their personal information would remain confidential and they hold the right to withdraw from the study whenever they wish to.

### Outcome measures

A self-administered questionnaire (Additional file) was developed after a thorough review of the literature of previously published papers regarding SARS-COV, MERS-COV, and SARS-COV-2 and following WHO & CDC guidelines. We used 70% as a cut-off value for all the questionnaires based on the previously published studies [15, 18, 19]. It consisted of both multiple-choice questions and Likert scale items. We made a short item questionnaire because too long questions might affect the quality of the study. After the development of the questionnaire, a validity and reliability test was performed. The prepared questionnaire was first sent to 5 randomly selected experts from medicine, nursing, and paramedics to give their feedback regarding its contents, simplicity, and significance. Secondly, a pilot study was carried out involving 40 individuals from all the respective departments. A Cronbach’s alpha of 0.74 was obtained.

The questionnaire was divided into 4 parts consisting of 30-items, demographic characteristics (10-items), knowledge (10-items), attitude (5-items) and practice (5-items). Demographic characteristics consisted of 10 items, including age, gender, level of education, marital status, profession, work experience, source of information, place of work, infection prevention and control (IPC) training, and online course regarding COVID-19. Knowledge consisted of 10 items, including causative agent, incubation period, mode of transmission, main symptoms, confirmatory diagnosis, high-risk population for severe outcome, preventive measures, current management option, possible complications and mortality rate. It was a multiple-choice question. The correct answer was given 1 point, and an incorrect answer was given 0 point. The score ranged from 0 to 10. Higher scores denoted better knowledge. While knowledge ≥7 is considered as adequate knowledge, <7 is considered as inadequate knowledge.

Attitudeconsisted of a 5-points Likert scale having 5-items, including worry about transmitting the virus to family, friend & society, belief of virus transmission from an asymptomatic patient, belief of IPC from hand-washing with soap & water, belief of development of a vaccine for COVID-19 and belief that COVID-19 would be controlled completely. Participants’ response was from 5 to 25. Higher scores denoted a better attitude. As per 70% cut-off points, it would become 17.5, but there was no score in the decimal so, a score of ≥18 was rated as a positive attitude, and ≤ 17 was rated as a negative attitude. The statement for options strongly agree, agree, neutral, disagree and strongly disagree was scored as 5, 4, 3, 2, and 1, respectively. The scoring system was just the opposite for worry about transmitting the virus to family, friend & society.

The practice also consisted of 5-points Likert scale having 5-items, including implementation of 5 moments of hand hygiene with 7 steps, utilization of 60% alcohol-based hand sanitizer in the absence of soap & water, wearing of PPE, carefully doffing of PPE and isolation of suspected or infected patients. Participants’ response was from 5 to 25. Higher scores denoted better practice. A similar scoring system obtained for the practice as a score of ≥18 was rated as appropriate, and ≤17 was rated as inappropriate practice. The statement for options was always, often, sometimes, rarely, and never are scored as 5, 4, 3, 2, and 1, respectively.

### Data management and analyses

We used Microsoft Excel 2016 and Statistical Package for Social Sciences (SPSS) version 23 for data analyses. One-way ANOVA and Chi-square or Fisher Exact tests were used to analyze continuous data and categorical data, respectively. The descriptive statistical analysis was completed for demographic variables. Continuous data were presented as mean ± SD, whereas categorical data were presented as number (n) or percentage (%). Pearson correlation analysis was used to investigate the relationship between knowledge, attitude & practice among F-HCWs. Binary logistic regression analyses were performed to find the factors associated with adequate knowledge, positive attitude, and appropriate practice, and the associations were expressed as adjusted Odds Ratio (aOR) and 95% Confidence Interval (CI). A *P*-value of < 0.05 was considered statistically significant.

## Results

### Demographic characteristics of participants (Table 1)

A total of 603 F-HCWs were involved in this web-based survey. Of them, 64.7% of the participants were aged 18 to 29 years, and the majority were female (71%). Nearly half of the participants (50.9%) were bachelor’s degree holders and single (51.6%). The vast majority of the participants were nurses (56.7%), and a higher proportion (36.3%) of participants had more than 5 years of work experience. Participants gathered information from various sources, where social media was much used. More than half of the participants (55.5%) represented the private hospitals. Only 43.8% of the participants were involved in the IPC training, and only 28.5% of the participants received the online courses regarding COVID-19.

**Table 1:** Demographic Characteristics of the Participants

Variables	Sample (***n*** = 603)	Percentage (%)
**Age**		
18–29 years	390	64.7
30–49 years	206	34.2
50+ years	7	1.2
**Gender**		
Male	173	28.7
Female	428	71
Others	2	0.3
**Level of Education**		
Diploma Level	155	25.7
Bachelor’s Degree	307	50.9
Master’s Degree or Above	141	23.4
**Marital Status**		
Single	311	51.6
Married	286	47.4
Divorced or Widow	6	1
**Profession**		
Nurse	342	56.7
Doctor	158	26.2
Paramedics	103	17.1
**Work Experience**		
Less than 2 years	198	32.8
2 to 5 years	186	30.8
More than 5 years	219	36.3
**Source of Information**		
Social Media	524	43.5
Television	288	23.9
Official Websites	393	32.6
**Place of Work**		
Private Hospital	335	55.5
Government Hospital	165	27.4
Semi-government or University hospitals	103	17.1
**IPC Training**		
Yes	264	43.8
No	339	56.2
**Online Course**		
Yes	172	28.5
No	431	71.5

*n* Number of participants, *WHO* World Health Organization, *CDC* Centers for Disease Control & Prevention, *IPC* Infection Prevention and Control

### Perceived level of knowledge, attitude and practice among F-HCWs (Table 2)

#### Knowledge

A total of 76% of the F-HCWs reported adequate knowledge in this survey. Statistically significant differences regarding the perceived level of knowledge among F-HCWs were observed among independent variables, including age, gender, level of education, marital status, profession, work experience, source of information, IPC training, and online course (*p* < 0.05). A higher proportion of adequate knowledge was noticed among the F-HCWs aged 50 + years (85.7%), males (89%), master’s degree or above holders (89.4%), divorced or widow (83.3%), doctors (89.2%), more than 5 years of work experience (84%), social media users (86.7%), participants working at the government hospitals (81.8%), and participants who received IPC training (81.4%) and online courses related to COVID-19 (82.6%).

**Table 2:** Chi-square test results of demographic variables vs. KAP

Variables		Knowledge			Attitude			Practice		
		Adequate (n, %)	Inadequate (n, %)	***P***-value	Positive (n, %)	Negative (n, %)	***P***-value	Appropriate (n, %)	Inappropriate (n, %)	***P***-value
**Age (**years)	18–29 years	278 (71.3)	112 (28.7)	.002	198 (50.8)	192 (49.2)	.012	311 (79.7)	79 (20.3)	.255
	30–49 years	174 (84.5)	32 (15.5)		126 (61.2)	80 (38.8)		158 (76.7)	48 (23.3)	
	50+ years	6 (85.7)	1 (14.3)		6 (85.7)	1 (14.3)		7 (100)	0 (0)	
**Gender**	Male	154 (89)	19 (11)	.000	105 (60.7)	67 (39.3)	.044	129 (74.6)	44 (25.4)	.161
	Female	303 (70.8)	125 (29.2)		223 (52.1)	205 (47.9)		345 (80.6)	83 (19.4)	
	Others	1 (50)	1 (50)		2 (100)	0 (0)		2 (100)	0 (0)	
**Level of Education**	Diploma Level	102 (65.8)	53 (34.2)	.000	70 (45.2)	85 (54.8)	.001	129 (83.2)	26 (16.8)	.235
	Bachelor’s Degree	230 (74.9)	77 (25.1)		165 (53.7)	142 (46.3)		241 (78.5)	66 (21.5)	
	Master’s Degree or Above	126 (89.4)	15 (10.6)		95 (67.4)	46 (32.6)		106 (75.2)	35 (24.8)	
**Marital Status**	Single	219 (70.4)	92 (29.6)	.006	165 (53.1)	146 (46.9)	.374	240 (77.2)	71 (22.8)	.584
	Married	234 (81.8)	52 (18.2)		163 (57)	123 (43)		231 (80.8)	55 (19.2)	
	Divorced or Widow	5 (83.3)	1 (16.7)		2 (33.3)	4 (66.7)		5 (83.3)	1 (16.7)	
**Profession**	Nurse	242 (70.8)	100 (29.2)	.000	172 (50.3)	170 (49.7)	.001	285 (83.3)	57 (16.7)	.009
	Doctor	141 (89.2)	17 (10.8)		107 (67.7)	51 (32.3)		117 (74.1)	41 (25.9)	
	Paramedics	75 (72.8)	28 (27.2)		51 (49.5)	52 (50.5)		74 (71.8)	29 (28.2)	
**Work Experience**	Less than 2 years	135 (68.2)	63 (31.8)	.001	114 (57.6)	84 (42.4)	.160	157 (79.3)	41 (20.7)	.929
	2 to 5 years	139 (74.7)	47 (25.3)		91 (48.9)	95 (51.1)		145 (78)	41 (22)	
	More than 5 years	184 (84)	35 (16)		125 (57.1)	94 (42.9)		174 (79.5)	45 (20.5)	
**Source of Information**	Social Media	398 (86.9)	126 (86.9)	.004	290 (87.9)	234 (85.7)	.067	411 (86.3)	113 (89.0)	.746
	Television	229 (50.0)	59 (40.7)		167 (50.6)	121 (44.3)		230 (48.3)	58 (45.7)	
	Official Websites	314 (68.6)	79 (54.5)		227 (68.8)	166 (60.8)		313 (65.8)	80 (63.0)	
**Place of Work**	Private Hospital	246 (73.4)	89 (26.6)	.113	174 (51.9)	161 (48.1)	.230	263 (78.5)	72 (21.5)	.971
	Government Hospital	135 (81.8)	30 (18.2)		93 (56.4)	72 (43.6)		131 (79.4)	34 (20.6)	
	Semi-government or University hospitals	77 (74.8)	26 (25.2)		63 (61.2)	40 (38.8)		82 (79.6)	21 (20.4)	
**IPC Training**	Yes	215 (81.4)	49 (18.6)	.005	156 (59.1)	108 (49.8)	.057	217 (82.2)	47 (17.8)	.083
	No	243 (71.7)	96 (28.3)		174 (51.3)	165 (48.7)		259 (76.4)	80 (23.6)	
**Online Course**	Yes	142 (82.6)	30 (17.4)	.017	109 (63.4)	63 (36.6)	.007	150 (87.2)	22 (12.8)	.002
	No	316 (73.3)	115 (26.7)		221 (51.3)	210 (48.7)		326 (75.6)	105 (24.4)	

*KAP* Knowledge, attitude and practice, *n* Number of participants, *WHO* World Health Organization, *CDC* Centers for Disease Control & Prevention, *IPC* Infection Prevention and Control

#### Attitude

Only 54.7%of the F-HCWs demonstrated a positive attitude. Statistically significant differences regarding the attitude among F-HCWs were observed among independent variables, including age, gender, level of education, profession, and online course (*p* < 0.05). A higher proportion of positive attitude was reported by the F-HCWs aged 50+ years (85.7%), other gender (100%), master’s degree or above holders (67.4%), married (57%), doctors (67.7%), less than 2 years of work experience (57.6%) social media users (87.9%) participants working at semi-government or university hospitals (61.2%) and participants who received IPC training (59.1%) and online course related to COVID-19 (63.4%).

#### Practice

Approximately 78.9% of the F-HCWs implemented appropriate practice. Only 2 independent variables, including the profession and online course, showed statistically significant differences with practice (*p* < 0.05). A higher proportion of appropriate practice was noticed among the F-HCWs aged 50+ years (100%), other gender (100%), diploma degree holders (83.2%), divorced or widow (83.3%), nurse (83.3%), more than 5 years of work experience (79.5%), social media users (86.3%), participants working at semi-government or university hospitals (79.6%), and participants who received IPC training (82.2%) and online courses related to COVID-19 (87.2%).

### Correlational analysis between knowledge, attitude and practice among F-HCWs (Table 3)

In this survey, overall average knowledge was 7.41 ±1.43, attitude was 17.60 ± 1.97, and practice was 20.72 ± 3.72 regarding COVID-19 among F-HCWs in Nepal. Pearson correlation analysis showed a significant correlation between knowledge, attitude and practice at the level of *p* = 0.01.

**Table 3:** Correlation analysis of Knowledge, Attitude and Practice

		Knowledge	Attitude
Attitude	Pearson Correlation	.112^**^	
	Sig. (2-tailed)	.006	
	N	603	
Practice	Pearson Correlation	.138^**^	.241^**^
	Sig. (2-tailed)	.001	.000
	N	603	603

**. Correlation is significant at the 0.01 level (2-tailed)

### Factors associated with adequate knowledge, positive attitude and appropriate practice towards COVID-19 (Table 4)

The factors significantly associated with adequate knowledge were male gender (aOR: 3.66; 95% CI: 1.97–6.82, *p* < 0.05), nurse and doctor (aOR: 2.10; 95% CI: 1.18–3.72 *p <* 0.05), websites (aOR: 1.83; 95% CI1.13–2.97 *p <* 0.05) and IPC training (aOR: 1.53; 95% CI: 1.02–2.31 *p* < 0.05). Similarly, factors significantly associated with positive attitude was online course related to COVID-19 (aOR: 1.49; 95% CI: 1.02–2.17, *p* < 0.05) only. Moreover, factors significantly associated with appropriate practice were master’s degree or above (aOR: 0.56; 95% CI: 0.31–1.00, *p <* 0.05) and online course related to COVID-19 (aOR: 2.43; 95% CI: 1.44–4.09, *p <* 0.05).

**Table 4:** Factors associated with knowledge, attitude and practice among frontline healthcare workers

Variables	Parameters	Knowledge	Attitude	Practice
		aOR (95% CI)	aOR (95% CI)	aOR (95% CI)
**Age**	18–29 years	1	1	1
	30 years or more	0.80 (0.42–1.53)	1.37 (0.81–2.32)	0.84 (0.43–1.62)
**Gender**	Female & Others	1	1	1
	Male	3.66 (1.97–6.82) *	1.24 (0.8–1.91)	0.87 (0.52–1.44)
**Marital Status**	Single & Divorced or Widow	1	1	1
	Married	1.31 (0.79–2.15)	0.90 (0.60–1.36)	1.68 (0.98–2.89)
**Level of Education**	Diploma & Bachelor’s degree	1	1	1
	Master’s degree or above	1.95 (0.99–3.81)	1.54 (0.94–2.52)	0.56 (0.31–1.00) *
**Profession**	Paramedics	1	1	1
	Nurse & Doctor	2.10 (1.18–3.72) *	1.43 (0.89–2.30)	1.57 (0.92–2.68)
**Work Experience**	5 years or less	1	1	1
	More than 5 years	1.47 (0.87–2.49)	0.85 (0.56–1.30)	1.11 (0.65–1.88)
**Source of Information**				
**Social Media**	No	1	1	1
	Yes	1.23 (0.65–2.31)	1.38 (0.80–2.37)	0.74 (0.37–1.47)
**Television**	No	1	1	1
	Yes	1.07 (0.67–1.72)	1.02 (0.68–1.53)	1.17 (0.72–1.92)
**Official Websites**	No	1	1	1
	Yes	1.83 (1.13–2.97) *	1.49 (0.96–2.27)	0.99 (0.59–1.65)
**Place of Work**	Private Hospital	1	1	1
	Government Hospital & Semi-government or University hospitals	1.22 (0.81–1.84)	1.23 (0.87–1.73)	1.10 (0.73–1.67)
**IPC Training**	No	1	1	1
	Yes	1.53 (1.02–2.31) *	1.29 (0.92–1.81)	1.27 (0.83–1.92)
**Online Course**	No	1	1	1
	Yes	1.56 (0.97–2.51)	1.49 (1.02–2.17) *	2.43 (1.44–4.09) *

*aOR* Adjusted Odds ratio, *CI* Confidence interval, *IPC* Infection Prevention and Control; *: Significant at *P* ≤ 0.05

## Discussion

It is a web-based national survey involving 603F-HCWs working at different hospitals or clinical settings. The vast majority of the participants were female, nurse, and from the private hospital. In this survey, F-HCWs reported adequate knowledge with a positive attitude and adopted the appropriate practice. Factors associated with adequate knowledge were male gender, nurse and doctor, source of information as websites and IPC training, whereas only online course was associated with a positive attitude; similarly, factors associated with the appropriate practice were master’s degree or above and online course.

In this survey, around 76% of F-HCWs illustrated adequate knowledge regarding COVID-19. However, it is relatively higher than the previous study conducted in Uganda [11]. There was only 69% sufficient knowledge and a bit lower than a Chinese study conducted by Zhang et al. [13] in Henan China, where 89% of the participants showed sufficient knowledge regarding the COVID-19. Moreover, a similar result was reported by a previous study carried out on MERS among F-HCWs of Saudi Arabia [15]. There are variances in the knowledge of F-HCWs while battling the outbreak in different countries. It could be due to differences in the cut-off points. Olum et al. [11] used 80% as a cut-off point to determine the level of knowledge, whereas Khan et al. [15] and we used 70%. In Zhang et al.’s study [13], the reason for a higher proportion of knowledge was probably due to better preparedness for the worst. It might be because the Henan province is a neighbouring province and was one of the severely affected places next to Wuhan.

Papagiannis et al. [17] reported that the high level of knowledge was significantly associated with positive attitude and practice among F-HCWs. We also had a similar correlation between knowledge, attitude and practice. However, previous studies have reported varying levels of perceived knowledge, attitude, and practice among the F-HCWs. Some studies have reported that the male participants to have a significantly higher knowledge [11]. In contrast, some have reported that female participants have higher knowledge than their male counterparts [12]. Some studies have reported that doctors have better knowledge and a positive attitude than others [13]. However, some reported the nurses to have better practice [14]. In this survey, male participants, doctors, having a master’s degree or above showed better knowledge and a positive attitude. Despite having a higher level of education, the F-HCWs did not show any appropriate practice. The reasons for inappropriate practice could be associated with the training they get, the working environment they have, etc. Because most of our hospitals did not provide sufficient sanitizers, PPEs, and even some hospitals, deducted the regular salary of the staff in such a harsh situation.

IPC training and online courses for F-HCWs are essential to update their knowledge and play a vital role in infection prevention [20]. WHO has started training sessions and online classes regarding prevention and control of COVID-19 to increase awareness and preparedness for the F-HCWs [21]. Adequate knowledge, positive attitude, and appropriate practice were present in the participants who attended IPC training and online courses. However, the knowledge was higher in those participants who took IPC training but was not associated with a positive attitude and appropriate practice. Nevertheless, those who took online courses regarding COVID-19 had a positive attitude and appropriate practice. Such results may be due to the fact that training is often organized by the institutions, and irrespective of their interest, participants are requested to attend the training. However, online courses are taken by those who have real interest, enthusiasm, and motivation towards the disease. So, they not only give full attention but also try to implement it in their daily activities.

Globally, thousands of F-HCWs are already infected, and hundreds have lost their lives. The risk of COVID-19 infection among F-HCWs is higher than the general population [8]. Among the countries, the United States has reported 16%, the Netherlands has 19.6%, Italy has 20%, and Spain has 26% infection rate among F-HCWs [17, 22]. Inadequate knowledge increases the risk of infection and might jeopardize their and their family’s lives. So, continuous knowledge update for this ever-changing pandemic by adequate training or course is crucial for the F-HCWs to tackle the COVID-19. The national and international authorities are continuously providing updates regarding COVID-19. However, social media was the most commonly used source of information than official websites (Ministry of Health and population, WHO, and CDC). Although the F-HCWs reported adequate overall knowledge, there were still knowledge gaps among different groups. Our participants mostly used online social media, radio, and television to gather knowledge, and they reported correct answers regarding the symptoms and prevention. Still, most of our participants were not sure about the confirmatory diagnosis as per the standard protocol. That is the defect of relying on only social media, not the standard source of information.

Despite having adequate knowledge, only 54.7% of F-HCWs had a positive attitude in this survey. Similarly, some previously published literature also revealed the lower rate of positive attitude by the F-HCWs in different countries [23, 24]. On the other side, a Chinese study [1] demonstrated a positive attitude among the majority population towards COVID-19. Perhaps, the Chinese were well trained and mentally prepared.

The vast majority of F-HCWs always fear infecting others, including their family, friends, and society [12, 15]. We also found a similar result of almost 88% of participants having a fear of infecting others while asking if they were worried about transmitting the virus to their family, friends & society. However, such fear is entirely normal and acceptable to help the F-HCWs to prepare for the worst during this grim and challenging situation. Despite having a fear of COVID-19, still, F-HCWs displayed appropriate practice. Hand hygiene practice is exceptionally essential. It is quite useful for infection prevention, and even the governments and many other stakeholders are promoting adequate hand hygiene practice. Saqlain et al. [25] reported that hand-washing to be one of the efficient barriers to disease transmission. In this survey, we asked the participants if they believed that hand-washing with soap and water was sufficient for infection prevention; 91.2% of the F-HCWs chose strongly agreed and agreed. Upon further asking if they are following 5 moments of hand hygiene with 7 steps, 88.4% of the participants reported often or always, but the remaining participants reported sometimes, rarely, and never. These results mean that our participants have appropriate hand hygiene practices. However, we still believe that hand hygiene is practically the most neglected procedure, and usually, F-HCWs do not follow all the moments and steps. Furthermore, multiple studies supported our findings of having appropriate practice [13, 18].

Unfortunately, the shortage of PPEs, such as facemask, face shield, gloves, goggles, and gown, during this COVID-19 crisis are the major problems faced by not only the developing countries like Nepal, but also the developed world like the USA, UK, and Italy. Being a developing country, an adequate supply of PPEs is a tremendous challenge in Nepal. Even if the government or local bodies supplied PPEs, especially the facemask and gown, the quality could not be assessed as most of them are prepared by local factories in an emergency situation. In this survey, only 56.4% of the participants were using PPEs. Most of them were using facemask, gown, and gloves. A negligible amount of N95 respirators were available for the F-HCWs. Albarrak et al. [18] reported that only 24.2% were wearing a facemask. It is essential to wear PPE throughout taking care of the patients, especially when performing aerosol-generating procedures such as; intubation, bagging, cardio-pulmonary resuscitation, and nebulization [22].. At the initial stage of the disease outbreak in Wuhan, many local F-HCWs were infected. Upon the arrival of the rescue team from different provinces, the infection to the F-HCWs went down to zero [16]. They mentioned for the infection transmission to the F-HCWs at the inial stage was carelessness, inadequate knowledge, insufficient PPEs, and even improper practice. So, the WHO has also given a particular focus on the correct use of PPEs, including masks, goggles, gloves, and gowns. Additionally, those F-HCWs who have used PPEs have comparatively fewer infection rates [26].

It is a fact that F-HCWs are highly susceptible to the infection, while their constant exposure makes them vectors for disease transmission [13]. Even though the F-HCWs have significant roles for infection prevention and disease transmission, it is necessary to follow strict rules of the PPE use, hand-hygiene, and isolation of the patients as per the CDC and WHO guidelines. The government and stakeholders have the responsibility of providing public awareness, regular updates of the infection prevention protocol, and provide adequate IPC training during this pandemic, and adequate logistic supply. Moreover, responsible bodies, including the government and hospitals, must focus on motivational factors, including the availability of the resources and provision of salary and incentives to the F-HCWs.

It is a cross-sectional survey, so we could not assess the changes. A web-based survey was conducted to identify the KAP of the F-HCWs working at different hospitals of the nation where they have full access to the internet service. We could not reach out to the place where this facility was not available. Despite these limitations, it is a national survey on the KAP regarding the COVID-19 among F-HCWs in Nepal. This survey would probably be responsible for providing up-to-date information and improve clinical practice among F-HCWs.

## Conclusion

F-HCWs reported adequate overall knowledge with a positive attitude and adopted the appropriate practice. The experienced F-HCWs with a higher level of education and who received IPC training and online courses regarding COVID-19 had better KAP. So, the stakeholders must arrange the educational programs and training for F-HCWs for better preparedness tackling with COVID-19. In addition, they must focus on motivational factors, including the availability of the resources and provision of salary and incentives to the F-HCWs.

## Supplementary Information

**Additional file 1.** Questionnaire to evaluate the Knowledge, Attitude and Practice among Frontline Healthcare Workers regarding COVID-19.

## Data Availability

The datasets supporting the conclusion of this article are included within the article. Upon a genuine request, raw data can be provided by the corresponding author.

## Abbreviations

CDC: Centers for Disease Control & Prevention
CMA: Community Medicine Assistant
COVID-19: Coronavirus Disease-2019
CT: Computed Topographic
F-HCW: Frontline Healthcare Worker
HA: Health Assistant
IPC: Infection Prevention and Control
KAP: Knowledge, Attitude and Practice
MERS-CoV: Middle East Respiratory Syndrome Coronavirus
PAHO: Pan American Health Organization
PPE: Personal Protective Equipment
RT-PCR: Reverse Transcription Polymerase Chain Reaction
SARS-CoV: Severe Acute Respiratory Syndrome Coronavirus
SPSS: Statistical Package for Social Sciences
WHO: World Health Organization
