# Supplementary material for: COVID-19: a National Survey on perceived level of knowledge, attitude and practice among frontline healthcare Workers in Nepal
Source: BMC Public Health. 2020 Dec 14;20:1905. doi: 10.1186/s12889-020-10025-8 (PMC7734608; doi:10.1186/s12889-020-10025-8)
Supplement: Supplementary file 1 — Additional file 1. Questionnaire to evaluate the Knowledge, Attitude and Practice among Frontline Healthcare Workers regarding COVID-19. [file 12889_2020_10025_MOESM1_ESM.docx]

**Additional Files**

*Questionnaire to evaluate the Knowledge, Attitude and Practice among Frontline Healthcare Workers regarding COVID-19*

**Part I: Demographic Characteristics of the Participants**

- 1. Age:
- 18 – 29 years
- 30 – 49 years
- 50+ years
  1. Gender:
- Male
- Female
- Others
  1. Level of Education :
- Diploma level
- Bachelor degree
- Master’s degree or above
  1. Marital status:
- Single
- Married
- Divorced / widow
  1. Profession:
     - Doctor
     - Nurse
     - Paramedics (Health Assistant or Community Medicine Assistant)
  2. Year of working experience:
- Less than 2 years
- 2 to 5 years
- More than 5 years
  1. Source of information (*multiple answers are possible*):
- Social media
- TV
- Official websites (Ministry of Health and Population, WHO, CDC)
  1. Place of work:
     - Private Hospital
     - Government Hospital
     - Semi-government or University Hospitals
  2. Did you participate in any infection prevention and control (IPC) training for the COVID-19 pandemic?
     - Yes
     - No
  3. Have you received any online courses regarding COVID-19?
- Yes
- No

**Part II. Knowledge, Attitudes & Practices (KAP) of Participants regarding COVID-19**

**Knowledge (**only single answer is possible**): -**

1. The virus causing the COVID-19 is…
   - SARS-CoV
   - MERS-CoV
   - SARS-CoV-2
   - Ebola Virus

The incubation period of the COVID-19 is…

- - 2 to 14 days
  - 2 to 11 days
  - 2 to 21 days
  - 7 to 21 days

1. The mode of transmission for COVID-19 is…
   - Respiratory droplet
   - Direct (infected person) & Indirect Contact (surface, objects such as thermometer)
   - Airborne Transmission (Aerosol)
   - All
2. The main symptom of COVID-19 is …
   - High-Grade Fever
   - Dry Cough
   - Shortness of Breath
   - All
3. Confirmatory diagnosis for COVID-19 is …
   - Rapid Diagnostic Test (RTD)
   - Reverse Transcription – Polymerase Chain Reaction (RT-PCR)
   - Chest Computed Tomography (CT) Scan
   - All
4. The high-risk population of COVID-19 for the severe outcome is …
   - Children
   - Pregnant Women
   - Elderly (over 65years) & People with underlying medical conditions such as; Diabetes, Heart disease, Kidney disease, Asthma, Cancer, etc.
   - All

Preventive measures for COVID-19 infection is ...

- - Hand-washing with soap & water
  - Social distancing and isolation of suspected & confirmed cases
  - Wearing of Personal Protective Equipment (PPE) while caring for infected patients
  - All

1. The management option for COVID-19 is …
   - Supportive & Symptomatic Management
   - Rehabilitative Management
   - Palliative Care
   - Anti-viral Therapy
2. Complications of COVID-19 is …
   - ARDS
   - Shock
   - Organ dysfunctions (Acute Kidney Injury, Acute Lung Injury, Acute Liver Injury, Acute Cardiac Injury, etc.)
   - All
3. The mortality rate of COVID-19 is…
   - 0.5 to 3%
   - 9.5%
   - 34.4%
   - 39%

**Attitude (***only single answer is possible***): -**

1. Being a frontline healthcare worker (F-HCW), having a high possibility of exposure, I am always worried about transmitting the virus to my family, friends & society.
   - Strongly Agree
   - Agree
   - Neutral
   - Disagree
   - Strongly Disagree
2. I believe that an asymptomatic patient can transmit the virus to others
   - Strongly Agree
   - Agree
   - Neutral
   - Disagree
   - Strongly Disagree
3. I believe that hand-washing with soap & water is sufficient for infection prevention
   - Strongly Agree
   - Agree
   - Neutral
   - Disagree
   - Strongly Disagree
4. I believe that the vaccine for COVID-19 will be developed
   - Strongly Agree
   - Agree
   - Neutral
   - Disagree
   - Strongly Disagree
5. I believe that COVID-19 will be controlled completely
   - Strongly Agree
   - Agree
   - Neutral
   - Disagree
   - Strongly Disagree

**Practice (***only single answer is possible***): -**

1. I am following 5 moments of hand hygiene with 7 steps
   - Always
   - Often
   - Sometimes
   - Rarely
   - Never
2. I am using 60% alcohol-based hand sanitizer in the absence of soap & water
   - Always
   - Often
   - Sometimes
   - Rarely
   - Never
3. I am wearing Personal Protective Equipment (PPE) while caring patient
   - Always
   - Often
   - Sometimes
   - Rarely
   - Never
4. I am carefully removing of Personal Protective Equipment (PPE) and discarding in the proper place
   - Always
   - Often
   - Sometimes
   - Rarely
   - Never
5. We are doing isolation of the suspected and/or infected patient
   - Always
   - Often
   - Sometimes
   - Rarely
   - Never
